# Adaptation in emergent adulthood for persons with prior experience in residential youth care in Norway: an observational study

**DOI:** 10.1186/s12889-026-28515-6

**Published:** 2026-07-10

**Authors:** Jan L. Wallander, Maria C. Stuifbergen, Stine Lehmann, Thomas Jozefiak, Hanne K. Greger

**Affiliations:** 1 Department of Psychological Sciences, University of California, Merced, USA; 2 Department of Mental Health, Faculty of Medicine and Health Sciences, Regional Centre for Child and Youth Mental Health and Child Welfare (RKBU Central Norway), NTNU – Norwegian University of Science and Technology, Trondheim, Norway; 3 Department of Clinical Psychology, Faculty of Psychology, University of Bergen, Bergen, Norway; 4 Department of Mental Healthcare – Emergency and Children, St. Olavs Hospital, Trondheim, Norway

**Keywords:** Adaptation, Emergent adulthood, Residential Youth Care, Longitudinal follow-up, Socioeconomic status, Civil status, Social relationships, Self-esteem, Quality of life

## Abstract

**Background:**

The adversities experienced by youth placed in Residential Youth Care (RYC) challenge their development through adolescence and into adulthood. Yet, we know little about how they adapt during the transition into emergent adulthood. Here, we examine their socioeconomic and civil status and psychosocial functioning, how they compare with peers, and whether there are systematic variations across sex and age.

**Methods:**

Ten years after the initial assessment of 400 12–20-year-olds residing in RYC in Norway, we conducted a follow-up assessment when they were ages 22–30, enrolling 157 (52% response rate). Participants completed an online survey where socioeconomic and civil status were measured with multiple-choice items. Psychosocial functioning in terms of social relationships, perceived social support, and self-perception was measured with standard psychometric scales and quality of life (QOL) with two commonly used ratings. Trained professionals rated global psychosocial functioning on a standard scale based on completion of a semi-structured psychiatric interview. Descriptive statistics were used to inform about the adaptation in these targeted areas. Sex differences were tested with Chi^2^ and t-tests and associations with age using linear or logistic regression. Results were compared to reference data representing the general population.

**Results:**

Compared to those in the general population, young adults with prior experience with RYC attained lower education, employment, and income. They became parents more often and at an earlier age. They also reported on average lower global self-worth and QOL, and for the majority their global psychosocial functioning was rated as in need of clinical services. However, the quantity and quality of their social relationships were reported similar to peers. Sex differences were few, other than females were more likely to be in intimate relationships and have a child. Adaptation was not better for older participants in the sample age range.

**Conclusions:**

Addressing the elementary question, “How are these vulnerable individuals doing in general in emergent adulthood?” results imply that the answer is “Not so good.” Social services need to consider how to improve interventions during childhood as well as in the transition when this group ages out of RYC to mitigate these negative long-term outcomes.

## Background

Most adolescents placed in residential youth care (RYC) have experienced significant psychosocial strains, which may include maltreatment [1], residential insecurity [2, 3], and interrupted education [4], and often drastic changes in their social environments [5]. Consequently, the majority of youth in RYC in Norway have one or more mental disorders, with a prevalence reaching up to 76% [6]. Their reported quality of life (QOL) and psychosocial functioning is also commonly impaired [7, 8]. International studies on youth in RYC confirm these findings e.g.,[ 9, 10]. These problems are likely to challenge the development of this group of youth through adolescence and into young adulthood [11]. Not surprisingly, a meta-analysis [12] reported high prevalence of mental disorders among adults who had involvement with child welfare services (CWS) or juvenile justice in childhood compared to adults in the general population. A recent study in Norway of young adults with prior experience with RYC reported a point prevalence of 78% for any mental disorder [13]. Other studies have found similarly high rates [e.g., 14–16]. These consistent findings thus suggest that the increased risk they carry from childhood continues into adulthood and can endure even into midlife [17].

In Norway, the process leading to RYC placement is managed by the municipal CWS [18]. Around 80% of children receiving services from CWS have interventions with their own family. The remainder are placed in CWS care, mainly in foster families. Placement in RYC is used only as a last option when other possibilities are not satisfactory [18], affecting only a small percentage who are predominately teenagers. In 2022 in Norway, 866 children and adolescents lived in RYC [19]. These are typically small units with 3 to 6 adolescents supervised by residential staff [18]. Because these are typically not treatment units, residents may receive treatment and follow-up from regularly available community services, such as child and adolescent mental health services (CAMHS) and other health and social services.

In Western culture, the path from dependency in childhood to independence in adulthood is now longer and more complicated than at previous points in history [20]. This has led to the view that this developmental period, generally considered as occurring between ages 18–29, is distinct and can be termed “emergent adulthood” to distinguish it from “full” adulthood [21]. At the end of this stage, most adults would live independently, are in long-term relationships, and have clear career paths. How they traverse emergent adulthood depends on the personal, family, and social resources they possess as they enter this stage, dynamic interactions between the emerging adult and their environment, and the support they receive during this stage [22]. Young adults who age out of RYC placement may be deficient in some or all these resources, challenging their successful adaptation to the expectations of adulthood.

Given these vulnerabilities, there is a need to illuminate how individuals who have prior experience with RYC adapt and function in emergent adulthood regarding the typical expectations for individuals in their 20s. How do individuals with RYC background progress in their general adult functioning spanning education and/or employment, financial independence, and marriage or intimate partnership? Moreover, how do they view themselves and their social relationships and quality of life? This information can provide valuable input to the discussion whether the investments in RYC lead generally to well-functioning adults in society. Whereas the mental health of this group has been well delineated, as summarized above, we are not aware of research into the general adaptation in emergent adulthood of individuals with prior experience with RYC. To fill this gap, we aim to address four questions in this research:

1. How do young adults with experience with RYC adapt to the expectations of emergent adulthood in terms of socioeconomic and civil status (education, employment, income, marriage/partnership, and children) and psychosocial functioning defined as experienced social relationships and support, self-perception, quality of life (QOL), and global functioning?
2. How does the adaptation during emergent adulthood of individuals with experience with RYC compare to that of similarly aged adults in the general population?
3. Are there sex differences in their adaptation?
4. Given that developments in adaptation should occur during emergent adulthood, are there indications of advancement across this period in the adaptation of young adults with experience with RYC?

## Methods

This research is part of the VINGO-study, a nation-wide prospective follow-up study examining the health and welfare of young adults with a history of living in RYC [23].

### Procedure

At baseline [6], youth ages 12–20 residing in RYC in Norway were eligible to participate with a few exceptions (unaccompanied minor without resident permit, acute crisis placement, insufficient proficiency in Norwegian). At follow-up 10 years later, all baseline participants were invited to take part in the VINGO-study in the period from January 2021 through April 2023 following procedures detailed elsewhere [13, 23]. They were invited to complete an online survey and a subsequent telephone interview, including the measures relevant to this study. Participants received a gift card (500 NOK = 44 EUR) after completing both assessments, and they were also included in a lottery for a larger value gift-card (10 000 NOK = 875 EUR).

### Sample

At baseline, from 601 eligible, 400 were enrolled (67% response rate, 58% females) in the age range 12 to 20 (age M = 16.8 years) [6, 24]. At their first out-of-home placement, they had a mean age of 12.5 years, and 59% reported that this first placement was in RYC. Extensive results based on the baseline data have been reported previously [e.g., 6–8, 24], showing among many details that 68% attended school, 12% were either in employment or in a training position for employment, and 18% were unemployed at that time. Moreover, 76% met criteria for a mental disorder and they reported poor QOL on average and a high prevalence of maltreatment experiences. All 400 participants provided written consent to be contacted for follow-up.

At follow-up 10 years later, contact was made with 302 (10 had died, 18 had no valid contact information, 70 did not respond despite repeated efforts), of whom 65 declined participation and 80 did not complete the consent form or survey, despite prompts. Details have been provided elsewhere [13] on recruitment and attrition, including the markedly higher mortality rate in this sample compared to expectations for this age range. This resulted in 157 young adults participating in the follow-up (52% response rate), with 68% female and mean age = 25.4 (range 22–30 years). Additional demographics are reported in the Results. Attrition analyses (details in [23]) indicated that VINGO participants were more often females (68% vs. 51%) and had a higher number of psychiatric disorders at baseline (M = 2.6 [SD 2.6] vs. 2.0 [SD 2.3]) compared to non-participants. Follow-up participants were also more likely to report suicide attempts and substance use problems at baseline compared to non-participants.

### Measures

#### Socioeconomic and civil status.

A series of questions in the online survey addressed socioeconomic and civil status, as detailed in Table 1.

#### Social relationships

Items drawn from ASEBA Adult Self-Report [25] were included in the online survey addressing how well the participant gets along with co-workers, supervisor at work, and marital/cohabitation partner (3-point rating scale: Not true = 0, Somewhat or sometimes true = 1, Very true or often true = 2); child(ren); siblings, mother, and father (3-point rating scale: Worse than average = 0, Variable or average = 1, Better than average = 2); and friends (4-point rating scale: Not as well as I like = 0 – Much better than average = 3). In addition, participants indicated how many close friends (not including family members) they have, as well as how often per month they had contact with any close friends and were visited by friends and family.

#### Perceived social support

A 5-item version of the Social Support Questionnaire (SSQ) [26], modeled after the original 27 item version [27], measured perceived social support. Each item asks to whom a person can turn in five hypothetical situations involving informational support, emotional support, and crisis intervention (details provide in [5]). Eight possible support persons (mother, father, boyfriend/girlfriend, sibling(s), friend(s), relative(s), neighbor(s), and others) are listed for each situation, together with the alternative “none.” In addition, satisfaction with the social support for each of the five hypothetical situations was measured on a 4-point Likert scale (Very poorly satisfied = 1 to Very satisfied *=* 4). Two scores are calculated from the SSQ: The Number (SSQ-N) score indicates the average number of supportive people that are identified across the five situations (range 0–8). The SSQ-Satisfaction (SSQ-S) score indicates the average satisfaction with the support received across the five situations (range 1–4).

#### Self-perception 

Global and domain-specific self-perceptions were measured with a revised Norwegian version [28] of the Self-Perception Profile for Adolescents (SPPA) [29]. Here we selected four of the original nine domains: Global Self-Worth, Close Friends, Physical Appearance, and Athletic Competence. Each domain contains five items, of which approximately half are reversed to avoid acquiescence response bias. The Norwegian version was culturally adapted, and the response format was simplified, where each statement was followed by four response options (Describes me poorly = 1 to Describes me very well = 4). The revised scale has shown good convergent and factorial validity [28].

#### Quality of life 

Quality of life (QOL) was measured with the WHOQOL-BREF developed by the World Health Organization [30]. For this study, we focus on the two global items as indicators of overall QOL, one asking, “How would you rate your quality of life?” (rated on a 5-point scale from Very poor = 1 to Very good = 5) and the other, ‘How satisfied are you with your health?” (rated on a 5-point scale from Very dissatisfied = 1 to Very satisfied = 5). The WHOQOL-BREF has been shown in numerous international studies to demonstrate good discriminant validity, content validity, and internal consistency and test–retest reliability [31].

#### Global psychosocial functioning 

The Global Assessment of Functioning-Functioning scale (GAF-F) [32] was used to measure overall psychosocial functioning. Administered as part of the telephone interview, the GAF-F is a numerical scale for rating the social, occupational, and psychological functioning of an individual, or how well one is meeting various problems in living. Scores range from 100 (extremely high functioning) to 1 (severely impaired). A score below 70 is generally suggestive of the need for clinical services, anchored by the description: “Some mild symptoms (e.g. depressed mood and mild insomnia) OR some difficulty in social, occupational, or school functioning (e.g., occasional truancy, or theft within the household), but generally functioning pretty well, has some meaningful interpersonal relationships.” For this study, the GAF-F was completed by trained mental health professionals based on a semi-structured psychiatric assessment conducted by telephone, during which time the Mini-International Neuropsychiatric Interview (MINI) [33] and the Norwegian version of the Posttraumatic Stress Disorder Checklist for DSM-5 [34] were administered. As detailed elsewhere [13], nine clinical psychologists and medical doctors with experience from mental health services and diagnostic competence were trained prior to conducting these interviews. In addition to providing diagnoses based on the participants’ answers during the interview (diagnostic results are reported elsewhere [13]), they completed the GAF-F at its conclusion.

### General population reference data

The results for young adults with prior experience in RYC were compared with data from the general population for selected outcomes using different sources. Comparison data regarding socioeconomic and civil status in 2023 (when the VINGO data collection was completed) were obtained for the national population from the publicly available StatBank Norway provided by Statistics Norway [19], the official governmental statistical agency in Norway.

For the SSQ and SPPA, reference data are from the sample drawn for the Young in Norway (YiN) study initiated in 1994 [35]. All Norwegian junior and senior high schools (students aged 12–19 years) were included in a register from which the schools were selected using cluster sampling. The sample was stratified according to geographical region, school size, and type to be representative of the student population in Norway [36]. Following the first wave of data collection with 12,287 participants, subsequent waves of data relevant to our study were collected in 1999 (T3), at ages 20 to 25, and 2005 (T4), at ages 25–30, with response rates of 84% and 82%, respectively [37]. The SSQ and SPPA (not including the domain Athletic Competence) were administered at both T3 and T4 data collections, providing descriptive statistics for comparisons with the VINGO sample.

Comparison results for the two WHOQOL-BREF items were drawn from a young adult community sample (*n* = 240, ages 20–30, 53% male) recruited in a British university city [38].

### User involvement

User representatives with lived experience from CWS or CAMHS contributed to the design of the VINGO study. They participated in meetings with the investigators where design and methods were discussed and in focus groups where questions for the survey and interview were tested.

### Analysis

Analyses were conducted using SPSS 30. Descriptive statistics are presented as frequency distributions for categorical variables and means and standard deviations for scale variables. Sex differences in the frequency distributions of categorical variables were examined using Chi^2^ and between the means of scale variables using t-tests. The associations with age were tested with linear or logistic regression as appropriate for the scale properties of the variables measuring adaptation.

### Ethics

The baseline study was approved by the Regional Ethical Committee for Medical and Health Research, Mid-Norway (REK) (2010/1965/REK Midt), and the VINGO study was approved by the Norwegian Agency for Shared Services in Education and Research (Sikt ref: 790618) and by REK (502016/REK Midt). At baseline, written informed consent was obtained from all participants. For youth under 16 years, a written informed consent from the guardian was obtained. At follow-up, all participants signed written informed consent before enrolment. The research was conducted in accordance with the Declaration of Helsinki.

## Results

### Socioeconomic status

Details are reported in Table 1. A plurality of 40% did not pursue education beyond 10th grade. In comparison, 21% in the general population at ages 20–24 and 18% at ages 25–29 have completed no more than a 10th grade education [19]. In our sample, 7% had earned a Bachelors or higher degree, compared to 25% in the general population at ages 20–24 and 51% at ages 25–29 [19]. With 18% in our sample currently enrolled in an educational program, 72% reported having had their education interrupted. Of those currently enrolled, a smaller portion (19%) were not satisfied with their educational program, in contrast to the 81% who were at least somewhat satisfied.

**Table 1:** Socioeconomic and civil status

Indicators	Response options	Total	Females	Males
		%	%	%
Education				
Highest education completed	High school < = 10th grade	40	42	38
	Vocational high school (1–2 yrs)	24	26	21
	Academic high school (3 yrs)	15	14	17
	Trade school/apprenticeship	14	10	23
	Bachelor’s degree	4	5	2
	Graduate degree	3	4	0
Education interrupted	Yes	72	73	69
	No	28	27	31
Plans for future education^a^	Manual/trade training	20	14	34
	University	30	31	28
	Do not know	57	59	53
Current education enrollment	Yes	18	20	12
	No	83	80	88
If enrolled, satisfied with current educational situation	Not true	19	24	0
	Somewhat/sometimes true	22	19	33
	Very/often true	59	57	67
Employment				
Gainful employement	Yes	48	43	58
	No	52	57	42
If employed, satisfaction with current employment	Not true	11	11	11
	Somewhat/sometimes true	36	40	30
	Very/often true	53	49	59
Income				
Household income all sources last year, before tax (NOK)	< 250,000	44	44	46
	250,000-450,000	33	32	33
	451,000-750,000	17	17	17
	751,000–1,000,000	4	4	4
	> 1,000,000	2	3	0
Public benefit recipient	Yes	58	62	50
	No	42	38	50
Type of public benefit received^a^	Work assessment allowance	24	29	14
	Disability 100%	22	21	24
	Social wellfare assistance	8	8	6
	Other programs	11	13	8
Dependent on family/partner financial support	Yes	29	33	20
	No	71	67	80
Satisfaction with personal economy	Very poor	17	15	22
	Poor	27	30	22
	Average	36	39	28
	Good	16	15	18
	Very good	4	1	10
Marriage/partnership				
Marital/cohabitation status	Never married/cohabitation	32	28	42
	Married/cohabitation	40	50	19
	Widow/er	1	0	4
	Seperated/divorced	3	3	4
	Other	23	20	31
Household living arrangement	Alone, no child(ren)	45	35	66
	Spouse/partner, no child(ren)	32	38	17
	Spouse/partner, with child(ren)	11	13	4
	Child(ren), no spouse/partner	4	6	0
	Other(s)	9	8	13
Romantic/sexual partner history	Yes now	63	72	43
	Yes previously	16	15	16
	No	21	12	41
Children				
Parent of child(ren)	Yes	29	34	19
	No	71	66	81
If parent, number of children	1	58	53	78
	2	31	36	11
	>=3	11	11	11

*NOK* Norwegian kroner

^a^Multiple responses could be selected

About one-half (47%) were gainfully employed currently. This compares to 70% in the population at ages 20–24 and 83% at ages 25–29 [19]. Among those employed in our sample, 11% were not satisfied with their current employment compared to 89% who were at least somewhat satisfied.

The reported median household yearly income level was 250,000-450,000 NOK. A plurality of 44% reported a household income of less than 250,000 NOK and 23% above 450,000 NOK. This compares with the median household yearly income in the Norwegian population of 416,000 NOK when the main income earner is less than age 25 and 739,800 NOK when the main income earner is ages 25–34 [19].

In the study sample, a majority of 58% were receiving public benefits of some type, including 24% who were receiving work assessment allowance, 22% full disability benefits, and 8% social welfare benefits. In the population of 22–30-year-olds in Norway, 5% received work assessment allowance, between 2% and 5% (depending on exact age) received disability benefits, and 5% received social welfare benefits [19].

Over two-thirds (71%) of our sample reported not receiving financial support from either family or a partner. Satisfaction with one’s personal economy was reported to be poor or very poor by 44%, average by 36%, and good or very good by 20%.

### Civil status

As also reported in Table 1, about one-third (32%) had never been married or cohabitating with a partner, in contrast with 40% who were married or cohabitating currently. In comparison, in the population, 21% of those aged 20–24 and 53% of those aged 25–29 were either married or cohabitating [19]. In our sample, 45% lived alone, 43% with a spouse or partner with or without children, and 13% in some other arrangement. Furthermore, 63% reported a current romantic or sexual partner and 16% had had one previously, whereas 21% reported no such relationship in their lifetime.

As shown in Tables 1 and 34% of women and 19% of men were parents, compared to 19% of women and 8% of men in the general population in the same age group [19]. Within this group of parents, 42% had 2 or more children. The median age at the birth of the first child was 22–24, and for 20% this occurred before age 19. In comparison, the mean age at the first child’s birth in the population was 30 years for females and 32 years for males [19].

### Social relationships

As detailed in Table 2. When asked about their relationships with other adults in their everyday environment, such as at work, school, and home, at least two-thirds reported it is Very or Often True that they get along well with others. Relationships with their children, when applicable, were reported to be Average or better by the vast majority (94%). The relationships were reported to be Variable or Average or better with one’s mother by 49% and with one’s father by 29%.

In comparison, relationships with close friends were rated by 92% as Average or better. The median number of close friends was 3–4. The number of contacts with close friends each month was 5 or more times for over two-thirds. Visits from friends or family occurred less frequently, however, with one-third reporting none in the last month.

**Table 2:** Social relationships

Variable	Response options	Total	Females	Males
		%	%	%
Get along well with…^a^				
Co-workers	Not true	0	0	0
	Somewhat/somtimes true	19	15	25
	Very/often true	81	85	75
Supervisor	Not true	11	11	11
	Somewhat/somtimes true	22	16	33
	Very/often true	67	73	57
Other students	Not true	0	0	0
	Somewhat/somtimes true	21	28	0
	Very/often true	79	72	100
Marital/cohabitation partner	Not true	5	5	8
	Somewhat/somtimes true	21	24	8
	Very/often true	73	71	85
Child(ren)	Worse than average	6	3	20
	Variable or average	34	32	40
	Better than average	57	62	40
Mother	Dead	9	11	4
	No contact	12	9	19
	Worse than average	30	32	25
	Variable or average	26	23	31
	Better than average	23	24	21
Father	Dead	21	21	20
	No contact	24	22	29
	Worse than average	26	31	16
	Variable or average	14	14	12
	Better than average	15	12	22
Close friends	Not as well as I like	8	9	6
	Average	12	13	10
	Better than average	25	24	27
	Much better than average	55	54	56
Number of close friends	0	5	6	4
	1	16	19	10
	2–3	46	46	45
	>= 4	33	30	41
Number of contacts each month with close friends	< 1	6	5	8
	1–2	12	13	10
	3–4	13	16	6
	>= 5	69	66	75
Number of visits each month from friends or family	< 1	32	28	40
	1–2	31	34	23
	3–4	18	19	15
	>= 5	19	18	21

^a^Percentage is based on responses for those for whom category is applicable

### Perceived social support

As shown in Table 3, the SSQ-N mean was 1.92, indicating the average number of supportive people that were identified across targeted situations. By comparison, the SSQ-N means were 2.70 at ages 20–25 and 2.79 at ages 25–30 for the reference sample of young adults in the Norwegian population [39]. In the study-sample, the mean satisfaction with the support received across the targeted situations (SSQ-S mean) was 3.25, corresponding closest to a rating of Satisfied with the support. This mean compares to that for the reference sample of 3.58 at ages 20–25 and 3.70 at ages 25–30. The mean SSQ-S ratings for the reference sample correspond closest to a rating of Very Satisfied. The difference between the means for the study and reference samples lies within the SD of the reference sample for the SSQ-N and SSQ-S scores, suggesting a lack of substantial difference, with one exception. The difference in the SSQ-S means when compared with the 25–30 age range in the reference sample exceeds the SD for the reference sample, with higher satisfaction reported by the reference sample.

**Table 3:** Scales measuring psychosocial adaptation

Variable	Response scale	VINGO Total M (SD)	VINGO Females M (SD)	VINGO Males M (SD)	Reference Sample M (SD)
Social Support Questionnaire^a^					
Number of supportive persons	0–8	1.92 (0.95)	1.95 (0.856	1.84 (1.13)	2.70 (1.07) 2.79 (1.13)
Satisfaction with support	1–4	3.25 (0.73)	3.31 (0.70)	3.12 (0.79)	3.58 (0.45) 3.70 (0.41)^c^
Self-Perceptions Profile for Adolescents^a^	1–4				
Global Self-Worth		2.32 (0.80)	2.30 (0.83)	2.38 (0.74)	2.97 (0.53)^c^ 3.02 (0.53)^c^
Close Friends		2.89 (0.81)	2.91 (0.85)	2.84 (0.74)	3.36 (0.57)^c^ 3.34 (0.58)^c^
Physical Appearance		2.33 (0.55)	2.28 (0.53)	2.44 (0.57)	2.75 (0.65) 2.77 (0.64)
Athletic Competence		2.02 (0.75)	1.85 (0.64)	2.34 (0.86)	N/A
WHOQOL^b^	1–5				
Overall quality of life		3.11 (1.09)	3.15 (1.07)	3.02 (1.15)	4.18 (0.73)^c^
Satisfaction with health		2.63 (1.13)	2.58 (1.13)	2.73 (1.15)	3.96 (0.79)^c^
Global Assessment of Functioning	0-100	59.51 (17.68)	58.44 (18.02)	62.00 (16.81)	

For each scale, a higher score reflects better functioning. N/A, not available from the Young in Norway study because it was not used at the follow-up assessments in young adulthood

^a^Reference sample data are drawn from the Young in Norway study (see Methods), reported for assessments conducted at ages 20–25 (top row) and 25–30 (bottom row) in each cell.

^b^Reference sample data are drawn from Skevington et al., 2014 (see Methods).

^c^The difference between the means for the VINGO Total and reference samples exceeds the SD of the reference sample, with higher score for the reference sample indicating better adaptation.

### Self-perceptions

The means for the global and domain-specific self-perceptions measured with the SPPA are detailed in Table 3. The difference between the means for the study and reference samples exceeds the SD of the reference sample for Global Self-Worth and the Close Friends domain, with the reference sample reporting higher self-perceptions.

### Quality of life

Overall QOL on WHOQOL-BREF was rated at a mean of 3.11, corresponding closest to a rating of Neither Poor nor Good. Satisfaction with Health was rated at a mean of 2.63, corresponding closest to a rating of Neither Satisfied nor Dissatisfied. These means compare to those of a reference sample of 4.18 and 3.96 for the Overall QOL and Satisfaction with Health items, respectively, which correspond closest to a rating of Good and Satisfied, respectively. The difference between the means for the study and reference samples exceeds the SD of the reference sample, with reference sample reporting higher Overall QOL and Satisfaction with Health. In our sample, 30% rated their Overall QOL as Poor or Very Poor compared to 8% who rated it as Very Good. Satisfaction with Health was rated by 50% as Dissatisfied or Very Dissatisfied, whereas 5% rated it as Very Good.

### Global psychosocial functioning

The mean rating by clinicians after a structured psychiatric interview using the GAF-F was 59.51. This mean lies in the range of ratings anchored by the descriptor “Serious symptoms…OR any serious impairment in social, occupational, or school functioning….” Moreover, 69% received a rating of 70 or below, which is generally suggestive of the need for clinical services. This included 16% who received a rating of 40 or below (anchored by “Some impairment in reality testing or communication…OR major impairment in several areas, such as work or school, family relations, judgement, thinking, or mood….”).

### Sex differences

Results for females and males for all variables measuring adaptation are reported in Tables 1, 2 and 3. When statistically tested only four variables yielded significant sex differences: Females were significantly more likely to be married or cohabitating compared to males (see Table 1; χ^2^ [5] = 18.39, *p* = .002) as well have a romantic or sexual partner currently (see Table 1; χ^2^ [2] = 17.25, *p* < .001). Females were also more likely to have a child (see Table 1: χ^2^ [2] = 5.78, *p* = .016). Finally, males reported higher Athletic Competence than females (see Table 3; *t*[147] = -4.33, *p* = .001). All other variables yielded non-significant sex differences.

### Age associations

As reported in Table 4, only two significant associations were found between age and the variables measuring adaptation, which indicated that with increased age, clinician-rated global functioning decreased (β = − 0.17, *t* = -2.06, *p* = .041) and the likelihood of being a parent increased (OR = 1.34, CI [1.06, 1.68]). All other variables yielded non-significant association with age.

**Table 4:** Regression modeling with age as independent variable

Dependent Variable	Association with Age
Linear Regression	β
Highest education completed	0.02
Household income all sources	0.16
Satisfaction with personal economy	− 0.13
Relationship with mother	− 0.05
Relationship with father	0.05
Relationship with close friend	0.01
Number of close friends	0.04
Number of contacts with close friends	0.02
Number of visits from friends or family	0.06
SSQ Number of supportive persons	− 0.03
SSQ Satisfaction with support	0.08
SPPA global self-worth	0.00
SPPA close friends	0.01
SPPA physical appearance	− 0.07
SPPA athletic competence	0.12
WHOQOL overall quality of life	− 0.11
WHOQOL health-	− 0.03
Global assessment of functioning	− 0.17*
**Logistic Regression**	**OR [CI]**
Gainful employment	1.02 [0.84, 1.25]
Public benefit	1.15 [0.94, 1.41]
Married/cohabitation	1.12 [0.90, 1.40]
Parent of child(ren)	1.34* [1.06, 1.68]

Dependent variables measured with continuous scales were tested with linear regression and those with categorical classification were tested with logistic regression

*SSQ*  Social Support Questionnaire, *SPPA*  Self-Perception Profile for Adolescents, *WHOQOL*  World Health Organization Quality of Life Group, *OR*  Odds ratio, *CI*  95th percentile confidence interval

*significant association with age (*p* < .05 for linear regression)

## Discussion

Young adults who age out of RYC placement may be deficient in the resources that support successful transition to emergent adulthood. Thus, there are concerns that their adult adaptation may be compromised. In this study we address the questions of how do individuals with RYC background progress in their general adult functioning spanning education and/or employment, financial independence, and marriage or intimate partnership, and how do they view themselves and their social relationships and quality of life? Results indicate that young adults with experience with RYC in their adolescence evidence reduced general adaptation in emergent adulthood, at ages 22 to 30. Compared to those in the general population, these young adults attain lower education, employment, and income and become parents more often and at an earlier age. They also report on average lower global self-worth and QOL, and for the majority, their global psychosocial functioning was independently rated as in need of clinical services. One exception appears to be their social relationships, where the self-reported quantity and quality are, for the most part, similar to age mates. Yet, they report a lower self-worth in their close relationships than peers in the general population. Overall, there were few sex differences, the main exception being that females were more likely to be in intimate relationships and have a child. There was no support for the expectation that general adaptation was better for individuals who are older versus younger within the emergent adulthood period.

The low educational attainment for this group is especially concerning as we generally expect education to provide the basis for establishing a career that can support independence and good functioning in adulthood. In fact, completing 13 years of education was the only factor associated with no mental disorder in emergent adulthood for those in this sample who experienced mental disorder in adolescence [13]. A plurality of 40% in our sample had dropped out of school by10th grade and only 7% had earned a Bachelor’s degree or higher. This outcome is in line with previous reports on foster care alumni in Scandinavia, where 8% to 13% obtained a college degree by age 26 [39]. Taken together, these results indicate that, despite the strong emphasis on educational equity in Scandinavian welfare-models, the emerging adults leaving RYC remain a vulnerable group regarding socioeconomic fulfillment. Indeed, less than one-half report gainful employment, which is considerably lower than in the Norwegian population. Correspondingly, their median income is also lower, and the majority is receiving public benefits. Consequently, close to one half report poor satisfaction with their current personal economy.

Whereas cohabitation or marriage was reported at a similar prevalence as in the general population, 34% of females and 19% of males in this group are parents to one or more children. This is about double the prevalence for the same age range of the general population. Moreover, the median age for having a first child, at ages 22–24, is considerably earlier than customary in Norway. The observed earlier and higher childbearing, especially in the context of low education and employment, can be a risk factor for the future need of social services. Moreover, young adults with a background from living in RYC have experienced a high burden of childhood adversities including family dysfunction, emotional neglect, and physical abuse [1, 40]. Most have lacked satisfactory parental nurturing in their own childhood, which could interfere with their own parenting to the detriment of their children. Taken together, these findings may represent serious risk factors for the next generation.

The comparably low overall QOL found for young adults with a history of RYC is expected given previous findings of reduced QOL among adolescents in RYC [1, 8] and foster care [41], as well as among young adult students with CWS background [42]. Although one may argue that the reference group in the current study is somewhat less relevant due to expected higher educational status, our results are consistent with studies showing reduced QOL among young people with a CWS background, across subgroups and comparison groups.

The most positive finding for this group is that the vast majority report good and frequent relationships with close friends. They are also generally as satisfied with their social relationships as are young adults in the general population, despite their lower socioeconomic status and more mental health issues [13]. This can represent a strength in their general adaptation that could enable resilience during emergent adulthood. Contradicting this finding, they report lower self-esteem in their close friendships than young adults in the general population. Future research will need to discern further details about the nature and experience of their social relationships.

Given that we expect advancement towards adulthood during the emergent adulthood period [43, 44], we anticipated indications of better adaptation with increased age within this period. It is concerning then that in general we do not find better adaptation in older compared to younger individuals in this group. This non-finding is based on cross-sectional analysis rather than examination of individual development over time, which would be preferable. It may also be that our sample size did not allow for the discovery of developmental trends.

### Strengths and limitations

We are not aware of any prior study that has illuminated the general adaptation of emergent adults with experience in RYC during their adolescence. Yet, we acknowledge several limitations with this study. Even though we expanded significant efforts, we were able to recruit just over one-half of the eligible participants for this 10-year follow-up. Whereas we were able to establish contact with about 75% of those eligible, even after a decade, over one-third refused participation or did not complete the consent form or survey regardless of our multiple prompts. Nonetheless, as this is a high risk and difficult to reach population, a response rate of 52% of those eligible must be considered satisfactory [45].

To provide a suitable comparison for our results, we attempted to obtain reference data that reasonably matched the VINGO sample, but that was not always possible. First regarding the data from Statistics Norway, the response options and group classification did not always match exactly those used in our survey. The reference data regarding perceived social support and self-perceptions were collected in the YiN study some 20 years prior to the VINGO data. The reference data for the quality-of-life items were obtained from a smaller sample in a university community in the U.K. We have been unable to find other data for young adults in the community for this instrument. In both latter cases, these issues raise the question how representative the reference data may be of the current general population in Norway. Therefore, we present these reference data to provide a context for clarifying the results from young adults with prior experience in RYC, rather than attempting formal comparisons with statistical tests.

### Implications

Considering these findings, misgivings must be raised about the ability of many individuals with prior experience with RYC to manage the challenges of emergent adulthood and establish themselves as well-functioning adults who can contribute positively to their own families and communities. Our findings may question the implementation of RYC as currently applied as the intervention for youth who cannot be supported and treated in their own families. At least, these findings point to the urgent need to consider what further interventions beyond institutional care are required to adequately support healthy development once placed in RYC. At the same time, it is well known that interventions are typically more impactful when initiated as early as possible in children’s development. We argue that comprehensive assessment is needed early in the process when a child or adolescent is referred to CWS, followed by implementation of evidence-based individually tailored interventions prior to considering RYC placement [46]. For those with trauma experiences, for example, there is the potential for trauma-focused therapy to be impactful (e.g. [47, 48]), but further research is required into this possibility.

Given the troubling adaptation we see for this group, together with continuing mental health issues [13], there is a strong argument that more comprehensive support is needed for these individuals to make a better transition from RYC care. Continuity of care must be emphasized. In Norway such care can be offered up to age 25 to individuals with a CWS history, but only 18% received such help in 2023 [49] despite that it appears to be associate with better educational outcomes [50].

Continuity of care requires collaboration between CWS and both physical and mental health services. Screening for physical and mental problems should be obligatory and referral as appropriate. Given the frequent report of trauma in their history [7, 13, 23], screening for dissociative symptoms is indicated. Such screening assessment is crucial because problems related to trauma can still be successfully treated in this age group.

Furthermore, given the low educational attainment, poor economic situation, and poor global self-esteem and QOL, our results indicate that after care services should include a stronger focus on guiding individuals to complete education or obtain skills facilitating employment and financial independence. Individuals may benefit from a mentor in the transition from RYC to increase perceived social support and the chance of success, as well as assist the individual in contact with health and social services.

### Future research

Numerous directions for future research can be considered based on these findings. First, it will be informative to conduct further follow-up study of this sample, for example into their 30s. It is possible that, whereas adaptation is generally not satisfactory in their 20s, positive development may be delayed such that a better adaptation is apparent later. Another fruitful question to address is, what may account for differential development in emergent adulthood? Among factors that may moderate adaptation could be differences related to their placement in and experience with RYC, such as reason for and age of placement, transitions between RYCs, and experiences with additional services while in RYC. Previous baseline research has examined their perception of social support from family members, peers, and staff when in the RYC environment [5, 51], raising the potential that different experiences with social support may moderate their later adaptation. Given the high prevalence of trauma experiences in the history of individuals in RYC [7, 52], differences in the timing and type of such experiences may also moderate adaptation in emergent adulthood. We have reported on the association between completing the standard 13 years of education and better mental health in emergent adulthood [13]. Educational attainment, therefore, may moderate other adult outcomes.

## Conclusions

We are not aware of any prior research that has examined the general adaptation of young adults who have had prior experience with RYC. Our research therefore represents a unique contribution to the understanding of how a high-risk population is able to transition into adulthood, providing information on a broad set of indicators that address the elementary question, “How are these vulnerable young adults doing in general?” Unfortunately, results from our study imply that the answer is, “Not so good.” We see lower educational, employment, and economic attainment and concerns about their sense of self-worth, quality of life, and psychosocial functioning. Moreover, they may face challenges raising children given that they are more likely to become young parents while lacking experience from healthy family functioning.

## Data Availability

Data and material will be made available on request made to Hanne K. Greger at [hanne.k.greger@ntnu.no](mailto: hanne.k.greger@ntnu.no) .

## Abbreviations

RYC: Residential youth care
CWS: Child welfare services
CAMHS: Child and adolescent mental health services
NOK: Norwegian kroner
EUR: Euros
ASEBA: Achenbach System of Evidence Based Assessment
SSQ: Social Support Questionnaire
SSQ-N: SSQ number of supportive persons score
SSQ-S: SSQ satisfaction with support score
SPPA: Self-Perception Profile for Adolescents
QOL: Quality of life
WHOQOL: World Health Organization Quality of Life Group
BREF: WHOQL short form of the WHOQOL-100
GAF-F: Global Assessment of Functioning-Functioning scale score
MINI: Mini-International Neuropsychiatric Interview
DSM-5: Diagnostic and Statistical Manual, 5^th^ edition
YiN: Young in Norway study

## References

[1] Greger HK, Myhre AK, Lydersen S, Jozefiak T. Previous maltreatment and present mental health in a high-risk adolescent population. Child Abuse Negl. 2015;45:122–34. 26003821 10.1016/j.chiabu.2015.05.003

[2] Hu N, Gelaw YA, Katz I, Fernandez E, Falster K, Hanly M, et al. Developmental trajectories of socio-emotional outcomes of children and young people in out-of-home care–insights from data of Pathways of Care Longitudinal Study (POCLS). Child Abuse Negl. 2024;149:106196. 37149427 10.1016/j.chiabu.2023.106196

[3] Villodas MT, Litrownik AJ, Newton RR, Davis IP. (2016) Long-term placement trajectories of children who were maltreated and entered the child welfare system at an early age: Consequences for physical and behavioral well-being. J Pediatr Psychol. 2016;41:46–54. 10.1093/jpepsy/jsv031 PMC4902863 25834181

[4] O’Higgins A, Sebba J, Gardner F. What are the factors associated with educational achievement for children in kinship or foster care: A systematic review. Child Youth Serv Rev. 2017;79:198–220.

[5] Singstad MT, Wallander JL, Lydersen S, Wichstrøm L, Kayed NS. Perceived social support among adolescents in Residential Youth Care. Child Fam Soc Work. 2020;25(2):384–93.

[6] Jozefiak T, Kayed NS, Rimehaug T, Wormdal AK, Brubakk AM, Wichstrom L. Prevalence and comorbidity of mental disorders among adolescents living in residential youth care. Eur Child Adolesc Psychiatry. 2016;25(1):33–47. 25749933 10.1007/s00787-015-0700-x PMC4698296

[7] Greger HK, Myhre AK, Lydersen S, Jozefiak T. Child maltreatment and quality of life: A study of adolescents in residential care. Health Qual Life Outcomes. 2016;14:74. 27161357 10.1186/s12955-016-0479-6 PMC4862063

[8] Jozefiak T, Kayed NS. Self-and proxy reports of quality of life among adolescents living in residential youth care compared to adolescents in the general population and mental health services. Health Qual Life Outcomes. 2015;13:104. 26197764 10.1186/s12955-015-0280-y PMC4509467

[9] Bronsard G, Alessandrini M, Fond G, Loundou A, Auquier P, Tordjman S, Boyer L. The prevalence of mental disorders among children and adolescents in the child welfare system: A systematic review and meta-analysis. Med. 2016;2016(957):e2622. 10.1097/MD.0000000000002622 PMC4998603 26886603

[10] Bürgin D, Witt A, Seker S, d’Huart D, Meier M, Jenkel N, et al. Childhood maltreatment and mental health problems in a 10-year follow-up study of adolescents in youth residential care: A latent transition analysis. Dev Psychopathol. 2025;37(1):68–83. 37990404 10.1017/S0954579423001426

[11] Mulraney M, Coghill D, Bishop C, Mehmed Y, Sciberras E, Sawyer M, Efron D, Hiscock H. A systematic review of the persistence of childhood mental health problems into adulthood. Neurosci Biobehav Rev. 2021;129:182–205. 34363845 10.1016/j.neubiorev.2021.07.030

[12] Seker S, Boonmann C, Gerger H, Jäggi L, d’Huart D, Schmeck K, Schmid M. Mental disorders among adults formerly in out-of-home care: A systematic review and meta-analysis of longitudinal studies. Eur Child Adolesc Psychiatry. 2022;31:1963–82. 34169369 10.1007/s00787-021-01828-0 PMC9663399

[13] Greger HK, Kayed NS, Lehmann S, Jozefiak T, Lydersen S, Wichstrøm L, Fjukstad KK. Prevalence and comorbidity of mental disorders among young adults with a history of residential youth care–a two-wave longitudinal study of stability and change. Eur Arch Psychiatry Clin Neurosci. 2025;27:1–1. 10.1007/s00406-025-02007-x PMC12904880 40287873

[14] Gypen L, Vanderfaeillie J, De Maeyer S, Belenger L, Van Holen F. Outcomes of children who grew up in foster care: systematic–review. Child Youth Serv Rev. 2017;76:74–83.

[15] Kääriälä A, Hiilamo H. Children in out-of-home care as young adults: a systematic review of outcomes in the Nordic countries. Child Youth Serv Rev. 2017;79:107–14.

[16] Seker S, Boonmann C, d’Huart D, Bürgin D, Schmeck K, Jenkel N, et al. Mental disorders into adulthood among adolescents placed in residential care: A prospective 10-year follow-up study. Eur Psychiatry. 2022;65:e40. 35730184 10.1192/j.eurpsy.2022.30 PMC9280920

[17] Brännström L, Forsman H, Vinnerljung B, Almquist YB. The truly disadvantaged? Midlife outcome dynamics of individuals with experiences of out-of-home care. Child Abuse Negl. 2017;67:408–18. 27884505 10.1016/j.chiabu.2016.11.009

[18] Official Norwegian Reports/Norges Offentlige Utredninger. Med barnet hele vegen (With the child the whole way). Oslo, Norway: 2023:24. Available from https://www.regjeringen.no/contentassets/3448ea4c535f4d20bbbb1ef4e05fc994/no/pdfs/nou202320230024000dddpdfs.pdf. Cited 2024 Nov 20.

[19] Statistics Norway. StatBank Norway [internet]. Oslo Norway: Statistisk sentralbyrå. Available from https://www.ssb.no/en/statbank/. Cited 2025 Jan 5 – April 15.

[20] Arnett JJ. Emerging adulthood: What is it, and what is it good for? Child Dev Perspect. 2007;1(2):68–73.

[21] Arnett JJ. Emerging adulthood: A theory of development from the late teens through the twenties. Am Psychol. 2000;55(5):469–80. 10842426

[22] Wood D, Crapnell T, Lau L, Bennett A, Lotstein D, Ferris M, Kuo A. Emerging adulthood as a critical stage in the life course. In: Halfon N, Forrest CB, Lerner RM, editors. Faustman EM editors Handbook of life course health development. Cham, Switzerland: Springer; 2018. 123 – 43. 31314293

[23] Greger HK, Stuifbergen MC, Jozefiak T, Kayed NS, Lydersen S, Rimehaug T, et al. Young adults with a history of residential youth vare: A cohort profile of a hard-to-reach population. Int J Environ Res Public Health. 2024;21(11):1447. 39595714 10.3390/ijerph21111447 PMC11593612

[24] Kayed NS, Jozefiak T, Rimehaug T, Tjelflaat T, Brubakk A-M, Wichstrøm L. Psykisk helse hos barn og unge i barneverninstitusjoner (Mental health in children and adolescents in child welfare institutions). Trondheim, Norway: Norwegian University of Science and Technology (NTNU); 2015.

[25] Achenbach TM, Rescorla LA. Manual for the ASEBA Adult Forms & Profiles. For Ages 18–59: Adult Self-report and Adult Behavior Checklist. Burlington, VT, USA: Achenbach System of Empiricallay Based Assessment; 2003.

[26] Wichstrøm L, Hegna K. Sexual orientation and suicide attempt: a longitudinal study of the general Norwegian adolescent population. J Abn Psychol. 2003;112(1):144–51. 12653422

[27] Sarason IG, Levine HM, Basham RB, Sarason BR. Assessing social support: the social support questionnaire. J Personal Soc Psychol. 1983;44(1):127–39.

[28] Wichstraum L. Harter’s Self-Perception Profile for Adolescents: Reliability, validity, and evaluation of the question format. J Person Assess. 1995;65(1):100–16. 10.1207/s15327752jpa6501_8 7643294

[29] Harter S. Self-perception profile for adolescents: Manual and questionnaires., Denver. United States: University of Denver; 1988, 2012. Available from https://du.digication.com/susan-harter/self-perception-profiles

[30] WHOQoL Group. Development of the World Health Organization WHOQOL-BREF Quality of Life Assessment. Psychol Med. 1998;28:551–8. 9626712 10.1017/s0033291798006667

[31] Hawthorne G, Herrman H, Murphy B. Interpreting the WHOQOL-BREF: Preliminary population norms and effect sizes. Soc Ind Res. 2006;77:37–59.

[32] American Psychiatric Association. Diagnostic and statistical manual of mental disorders: DSM-IV. Washington, DC: American Psychiatric Association; 1994. pp. 25–35.

[33] Sheehan DV, Lecrubier Y, Sheehan KH, Amorim P, Janavs J, Weiller E, Hergueta T, Baker R, Dunbar GC. The mini-international neuropsychiatric interview (M.I.N.I.): The development and validation of a structured diagnostic psychiatric interview for DSM-IV and ICD-10. J Clin. Psychiatry. 1998:59 (Suppl. S20);22–33, quiz 34–57. 9881538

[34] Blevins CA, Weathers FW, Davis MT, Witte TK, Domino JL. The posttraumatic stress disorder checklist for dsm-5 (pcl-5): Development and initial psychometric evaluation. J Trauma Stress. 2015;28:489–98. 26606250 10.1002/jts.22059

[35] Norwegian Social Research NOVA. Young in Norway [internet]. Oslo, Norway: Oslo Metropolitan University. Available from https://ung-i-norge.no/en/. Cited 2025 April 1.

[36] Wichstrøm L. The emergence of gender difference in depressed mood during adolescence: the role of intensified gender socialization. Dev Psychol. 1999;35(1):232–45. 9923478

[37] Fluit S, Kunst JR, Bierwiaczonek K, von Soest T. Self-esteem trajectories over three decades predict opposition to social equality in midlife. Proc Natl Acad Sci. 2023;120(1):e2212906120. 36577060 10.1073/pnas.2212906120 PMC9910465

[38] Skevington SM, Dehner S, Gillison FB, McGrath EJ, Lovell CR. How appropriate is the WHOQOL-BREF for assessing the quality of life of adolescents? Psychol Health. 2014;29(3):297–317. 24192254 10.1080/08870446.2013.845668

[39] Vinnerljung B, Hjern A, Cognitive. educational and self-support outcomes of long-term foster care versus adoption. A Swedish national cohort study. Child Youth Serv Rev. 2011;33:1902–10.

[40] Åsen ME, Schalinski I, Lehmann S, Lydersen S, Von Oertzen T, Greger HK. Child maltreatment in young adults with residential youth care background: Prevalence and post-placement trends. Child Abuse Negl. 2024;157:107074. doi: 10.1016/j.chiabu.2024.107074 (Erratum in: Child Abuse Negl. 2025;161:107278. 10.1016/j.chiabu.2025.107278). 39395227

[41] Larsen M, Goemans A, Baste V, Wilderjans TF, Lehmann S. Predictors of quality of life among youths in foster care—a 5-year prospective follow-up study. Qual Life Res. 2021;30. 10.1007/s11136-020-02641-z. ,543 – 54. PMC7886817 32974880

[42] Lehmann S, Hysing M, Sivertsen B. Academic performance, health and support needs: Comparing foster care alumni and peers in higher education in Norway. Intl J Env Res Pub Hlth. 2024;21:1470. 10.3390/ijerph21111470. PMC11593920 39595737

[43] Messersmith EE, Schulenberg JE. Goal attainment, goal striving, and well-being during the transition to adulthood: a ten-year U.S. national longitudinal study. New Dir Child. 2010(130):27–40. 10.1002/cd.279 PMC4551495 21154829

[44] Pettit JW, Roberts RE, Lewinsohn PM, Seeley JR, Yaroslavsky I. Developmental relations between perceived social support and depressive symptoms through emerging adulthood: blood is thicker than water. J Fam Psychol. 2011;25:127–36. 10.1037/a0022320. 21355652 PMC3079557

[45] Wu MJ, Zhao K, Fils-Aime F. Response rates of online surveys in published research: A meta-analysis. Comput Hum Beh Rep. 2022;7:100206.

[46] Hiller RM, Lehmann S, Lewis SJ, Minnis H, Shelton KH, Tarren-Sweeney M, Taussig HN. Accommodating complexity: The need for evidence-informed mental health assessments for children in out-of-home care. J Amer Acad Child Adol Psychiatry. 2023;62(1):12–8. 10.1016/j.jaac.2022.06.021 35987299

[47] McGuire A, Steele RG, Singh A. Systematic review on the application of trauma-focused cognitive behavioral therapy (TF-CBT) for preschool-aged children. Clin Child Fam Psychol Rev. 2021;24(1):20–37. 33428071 10.1007/s10567-020-00334-0

[48] Siehl S, Robjant K, Crombach A. Systematic review and meta-analyses of the long-term efficacy of narrative exposure therapy for adults, children and perpetrators. Psychother Res, 2020; 31:695–710. 33205713 10.1080/10503307.2020.1847345

[49] Bufdir. Unge med passering som ettervernstiltak [Young people with placement as an aftercare measure]. https://www.bufdir.no/statistikk-og-analyse/barnevern/unge-med-ettervernstiltak/#section-richtext-13. Accessed 15 June 2025.

[50] Paulsen V, Wendelborg C, Riise A, Berg B, Tøssebro J, og, Caspersen J. Ettervern - en god overgang til voksenlivet? Helhetlig oppfølging av ungdom med barnevernerfaring [Aftercare - a good transition to adulthood? Holistic follow-up of youth with child welfare experience]. Trondheim: NTNU. 2020.

[51] Singstad MT, Wallander JL, Lydersen S, Kayed N. Perceived social support and symptom loads of psychiatric disorders among adolescents in residential youth care. Social Work Res. 2022;46(1):30–43.

[52] Greger HK, Myhre AK, Klöckner CA, Jozefiak T. Childhood maltreatment, psychopathology and well-being: The mediator role of global self-esteem, attachment difficulties and substance use. Child Abuse Negl. 2017;70:122–33. 28609692 10.1016/j.chiabu.2017.06.012

